# Effects of an artificial pancreas on postoperative inflammation in patients with esophageal cancer

**DOI:** 10.1186/s12893-024-02365-8

**Published:** 2024-03-02

**Authors:** Ryoko Miyauchi, Yuichiro Miki, Hiroaki Kasashima, Tatsunari Fukuoka, Mami Yoshii, Tatsuro Tamura, Masatsune Shibutani, Takahiro Toyokawa, Shigeru Lee, Kiyoshi Maeda

**Affiliations:** Department of Gastroenterological Surgery, Osaka Metropolitan University Graduate School of Medicine, 1-4-3, Asahimachi, Abenoku, Osaka, 545-8585 Japan

**Keywords:** Esophageal cancer, Artificial pancreas, Postoperative pneumonia

## Abstract

**Purposes:**

Subtotal esophagectomy for esophageal cancer (EC) is associated with high morbidity rates. Tight glycemic control using an artificial pancreas (AP) is one of the promising strategies to reduce postoperative inflammation and morbidities. However, the effects of tight glycemic control using AP in patients with EC are yet to be fully elucidated.

**Method:**

This study reviewed 96 patients with EC who underwent subtotal esophagectomy. The postoperative inflammation parameters and morbidity rates were compared between patients who used the AP (*n* = 27) or not (control group, *n* = 69). AP is a closed-loop system that comprises a continuous glucose monitor and an insulin pump.

**Results:**

The numbers of white blood cells (WBC) and Neutrophils (Neut) were noted to be lower in the AP group than in the control group, but with no significant difference. The ratio in which the number of WBC, Neut, and CRP on each postoperative day (POD) was divided by those tested preoperatively was used to standardize the results. The ratio of WBC and Neut on 1POD was significantly lower in the AP group than in the control group. The rate of surgical site infection was lower in the AP group than in the control group.

**Conclusion:**

AP significantly decreased WBC and Neut on 1POD; this suggests the beneficial effects of AP in alleviating postoperative inflammation.

**Supplementary Information:**

The online version contains supplementary material available at 10.1186/s12893-024-02365-8.

## Introduction

Patients with esophageal cancer have quite poor prognoses, partly because of the high rate of lymph node metastasis. One of the standard treatments is esophagectomy with a radical lymph node dissection. Surgical progress and postoperative management have improved both short- and long-term postoperative outcomes [1, 2]. However, esophagectomy remains associated with high morbidity and mortality rates [3, 4].

Surgical intervention including esophagectomy produces oxdative stress and inflammatory cytokines, which decreases insulin secretion and increase insulin resistance, resulting in hyperglycemia. This surgical stress induced hypergycemia could be a cause of postoperative infections due to leukocyte deficiency, granulocyte adherence, inpaired phagocytosis, delayed chemotaxis, and depressed bactericidal capacity. This suggests that glycemic control could be important for reducing inflammation after esophagectomy.

Randomized controlled trials showed that intensive insulin therapy (IIT) reduces organ failure and mortality, especially in the medical intensive care unit. Thus, theoretically, one of the possible strategies to decrease the morbidity rate after esophagectomy is intensive blood glucose control. However, hypoglycemia is the most significant drawback of IIT; in fact, the NICE-SUGAR trial reported increased mortality associated with IIT. These studies suggest normoglycemia (< 180 mg/dL) as a standard target for critically ill patients. Currently, not only blood glucose value but also glucose variability could be associated with mortality [5], but this issue remains to be fully investigated.

The artificial pancreas (AP) is a closed-loop system that comprises a continuous glucose monitor and an insulin pump; it makes tight glucose control possible with reduced glucose variability. The usefulness of AP in the surgical field has been reported in several studies [6–10]. However, the effects of AP on postoperative inflammation in patients who underwent esophagectomy are yet to be determined. Therefore, herein, we investigated the safety and efficacy of AP in terms of postoperative inflammatory parameters.

## Methods

### Patients

Herein, we reviewed 116 patients who underwent surgical treatments for esophageal cancer. Patients who underwent pharyngolaryngoesophagectomy and mediastinoscopic esophagectomy were excluded. As a result, 105 patients with esophageal cancer underwent subtotal esophagectomy with lymph node dissection at the Department of Gastroenterological Surgery, Osaka Metropolitan University Hospital, from January 2019 to January 2021. Any stages were included in this study. Neo-adjuvant chemotherapy (NAC) was routinely performed for patients with cStage II-III. 67 patients received NAC, and most often used regimen was DCF.

500 mg of Methylprednisolone was rountinely injected just before starting the operation.

During the operation, peri-esophageal, post-mediastinal, and supra-diaphragmatic lymph nodes were completely dissected concerning during the thoracic lymphadenectomy. The lymph nodes around the left and right recurrent laryngeal nerves in the upper thorax were then carefully dissected to preserve the nerves. These procedures were performed either by open, video-assisted, or robot-assisted methods. A gastric conduit was used for the reconstruction, and gastric mobilization was performed through hand-assisted laparoscopic surgery (HALS) or open laparotomy. With video assistance, the surgeon performed the HALS using the left hand through a 7-cm midline mini-laparotomy and two or three additional trocars. The conduit was pulled up to the neck through retrosternal routes [11, 12].

Pulmonary exercise with incentive spirometry was routinely performed as part of preoperative management. Smoking cessation was recommended for patients with smoking habits.

### Glycemic control and artificial pancreas

Conventional glycemic control was performed in the first half period (control group, *n* = 69). The blood glucose was checked every 2 h, and insulin infusion was performed when the blood glucose level exceeded 150 mg/dL. In detail, 2, 4, 6, 8, and 10 units of insulin was injected when the blood glucose level was “150–200”, “200–250”, “250–300”, “300–350”, and “over350”, respectively. This management was continued until the discharge of ICU (POD2).

Tight glycemic control by AP was achieved (AP group, *n* = 27) in the latter half of the period. AP (Nikkiso, Tokyo, Japan) uses a dual-lumen catheter blood sampling technique every 2 s and automatically infuses insulin and/or glucose to adjust blood glucose levels. The product name of AP is STG-55, and peripheral vein was used as vascular access. The target glucose range of 100–130 was maintained in the AP group. This management was also continued until the discharge of ICU (POD2).

Patients whose blood glucose was < 180 during AP usage were included in this analysis (*n* = 27). Blood glucose levels were noted to exceed 180 despite AP use during the same period in nine patients, who were then excluded from the main analysis (*n* = 96) as this study aims to determine the effects in patients whose blood glucose was well controlled by AP.

Clinical pathways were used for all included patients, so perioperative clinical managements were basically same in both groups.

### Data collection

Preoperative and perioperative factors were extracted from a prospective database maintained by our university hospital. The following preoperative factors were collected: age, sex, preoperative CRP concentration, white blood cells (WBC), Neutrophils (Neut), and clinical stage (cStage). The following intraoperative factors were collected: the type of thoracic and abdominal surgical procedure, operation time, and estimated blood loss. T and N were classified according to the seventh edition of the Union Internationale Contre le Cancer tumor lymph node metastasis classification system.

Data with regard to complications were collected, and their severity was rated based on the Clavien–Dindo classification [13]. Any grade was defined as present of complications. Informed consent was obtained from all participants by opt-out method. This study was approved by the Human Ethics Review Committee of Osaka Metropolitan University.

### Statistical analysis

The association between the AP and control groups and perioperative parameters was analyzed using *χ*^2^ test or t-test. All statistical analyses were performed using the JMP statistical software (version 10; SAS Institute, Cary, NC). Two-sided probability (*p*) values of < 0.05 were considered statistically significant.

## Results

### Baseline characteristics and representative data for glucose level

**Fig. 1:**
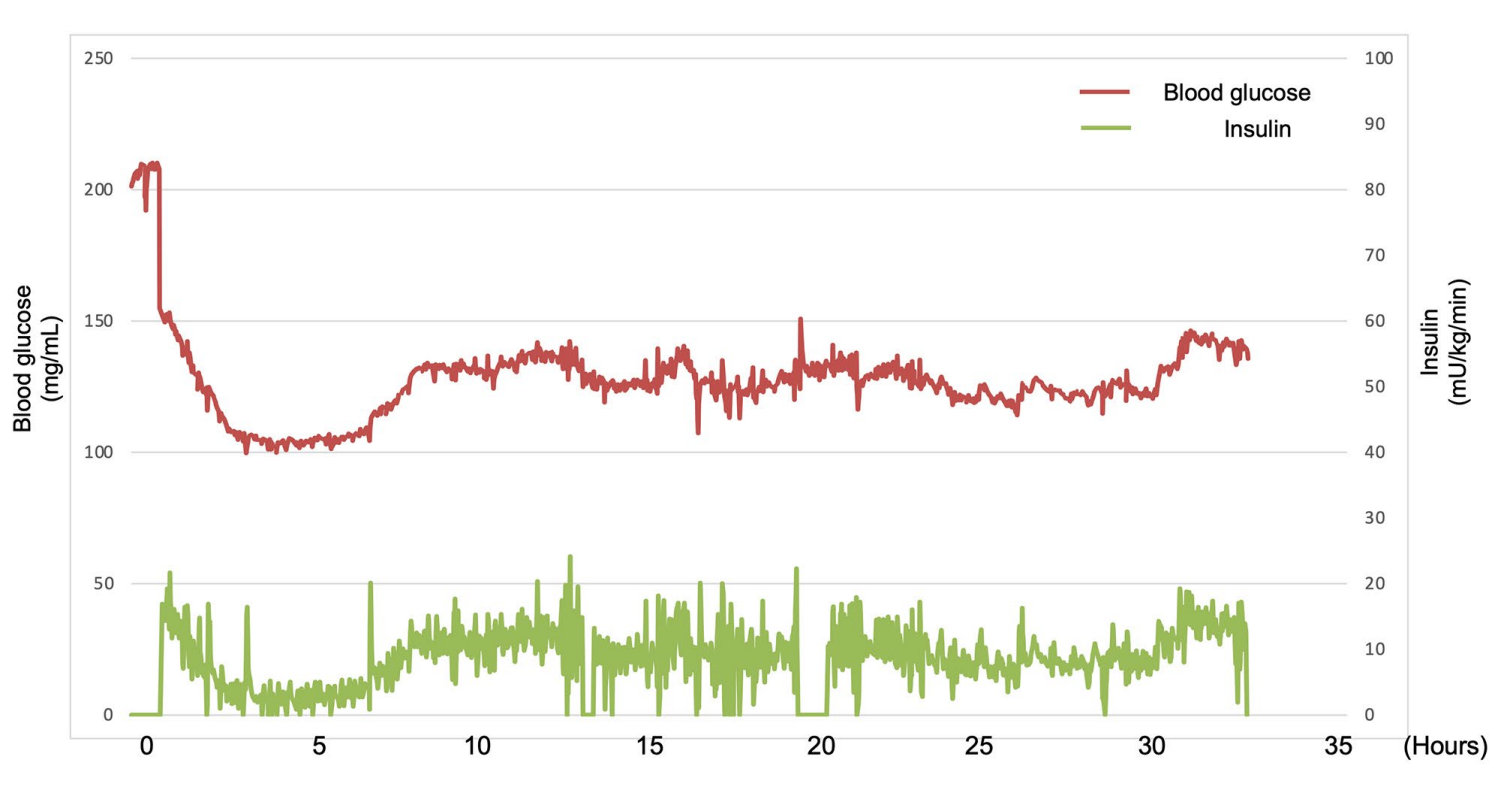
Representative data chart of blood glucose level in a patient who used AP.

**Fig. 2:**
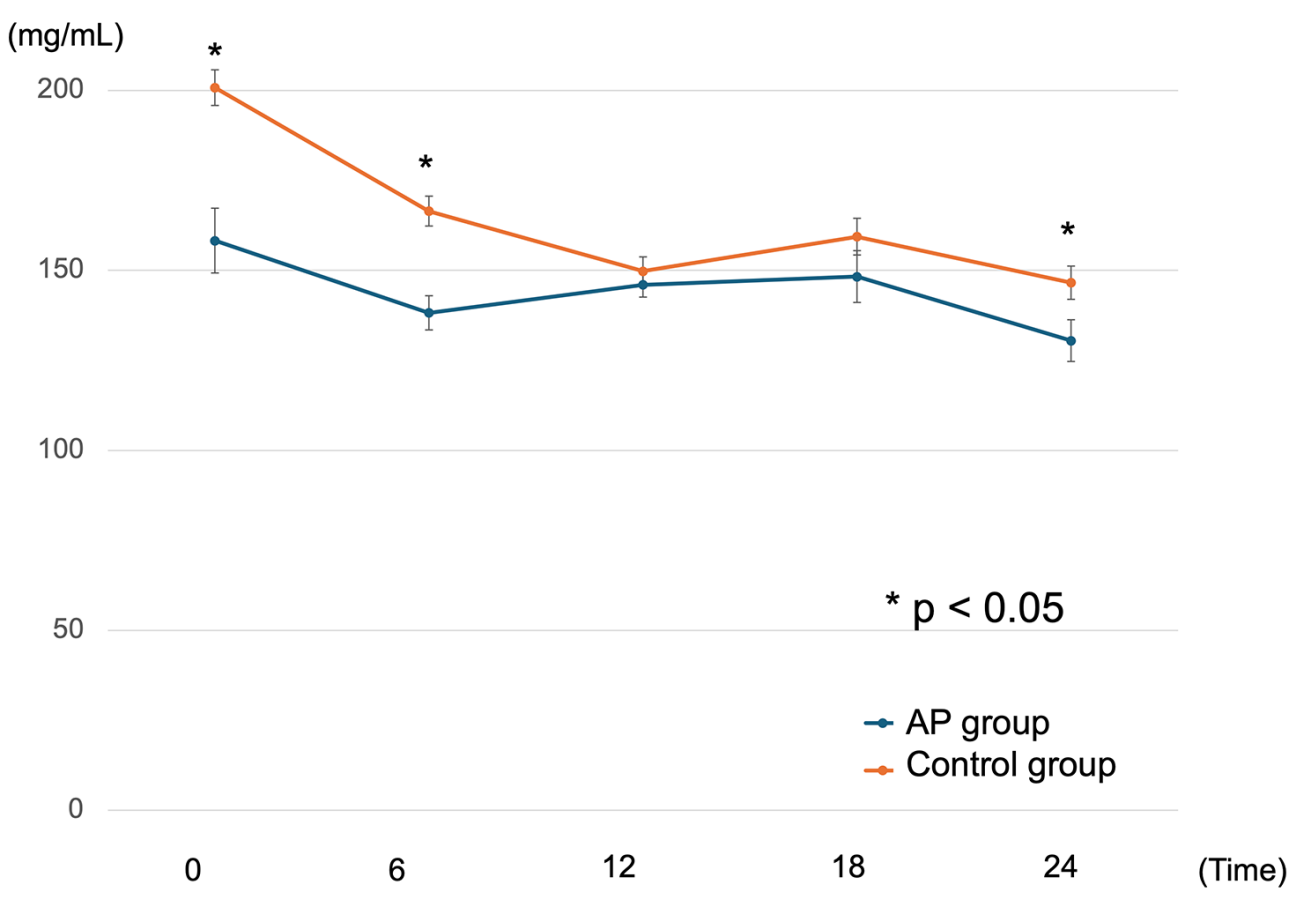
The average and standard error of blood glucose level on 1POD in AP and control group

Baseline patient characteristics in both groups are presented in Table 1. The median ages of patients in the AP and control groups were 67.1 and 68.5 years, respectively (*p* = 0.525), and > 70% of patients were male in both groups. Stages were well balanced between groups (*p* = 0.570). Video-assisted thoracic surgery and HALS were the most preferred operative procedures both in control and AP group. Seven patients (25.9%) of AP group and seven patients (10.1%) of control group underwent robotic surgery. Operation time was longer in the AP group than in the control group, but with no significant difference (589 vs. 558 min, *p* = 0.079). This is explained by the introduction of robotic surgery mainly in the latter half of the study period. Figure 1 shows a representative data chart of blood glucose levels in a patient who used AP. The blood glucose level is almost always approximately 125 (mg/ml). None of the study participants experienced hypoglycemia. The average and standard error of blood glucose level from 0 to 24 o’clock on postoperative day1 were shown in Fig. 2. Blood glucose level at 0, 6, and 24 o’clock were significantly lower in AP group than the control group. The influence of diabetes mellitus is shown in supplementary Fig. 1. Generally, blood glucose level is higher in DM patients than non-DM patients both in control and AP group.

**Table 1:** Patient characteristics and operative details in both groups

	AP group	control group	*p*-value
Age*	66 (47–86)	71 (46–90)	0.526
Sex			0.588
Male Female	20 (74.1%) 7 (25.9%)	55 (79.7%) 14 (20.3%)	
Diabetes Mellitus			0.503
Present Absent	2(7.4%) 25(92.6%)	9(13.0%) 60(87.0%)	
Neoadjuvant chemotherapy			0.031
Performed Not performed	14((51.9%) 13(48.1%)	52(75.4%) 17(24.6%)	
Tumor location			0.218
Ce Ut Mt Lt Ae	0 (0%) 5 (18.5%) 19 (70.3%) 3 (11.1%) 0 (0%)	1 (1.4%) 13 (18.8%) 33 (47.8%) 20 (28.9%) 2 (2.9%)	
Operative approach			0.094
VATS Robot Open	16(59.3%) 7(25.9%) 4(14.8%)	54(78.3%) 7(10.1%) 8(11.6%)	
Operation time (min) *	589 (468–722)	558 (393–958)	0.1397
Estimated blood loss (mL) *	262 (50–680)	341 (50-1640)	0.2608

Values in parentheses are percentages unless indicated otherwise; * Values are the medians (range)

### Effect of AP on inflammation markers

**Fig. 3:**
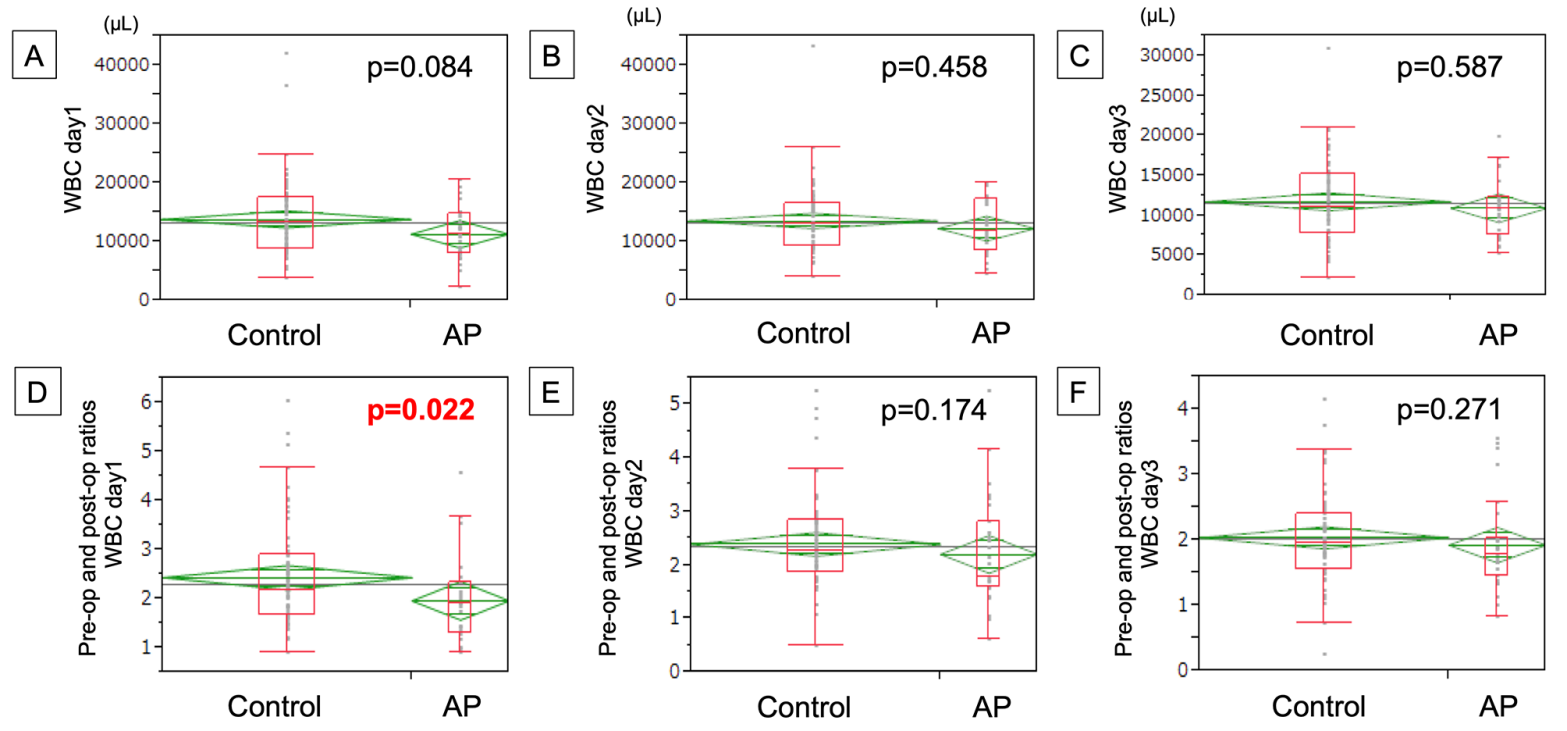
The WBCs count on 1st -3rd POD depending on the use of AP (**A-C**), and the ratio of WBCs where the number of WBCs on each postoperative day was devided by preoperative data (**D-F**)

Figure 3A–C show the WBC on postoperative days (PODs) 1, 2, and 3, depending on the use of AP. It was noted that AP tends to decrease the number of WBCs, especially on POD 1 (median: 11,300/µL [AP group] vs. 13,300/µL [control group]), but with no significant difference (*p* = 0.084). The ratio of WBCs (WBC ratio), wherein the number of WBCs on each postoperative day was divided by preoperative data, was evaluated to standardize the result (Fig. 3D–F). As per our findings, it was determined that the ratio of WBCs was significantly lower in the AP group than in the control group (*p* = 0.022).

**Fig. 4:**
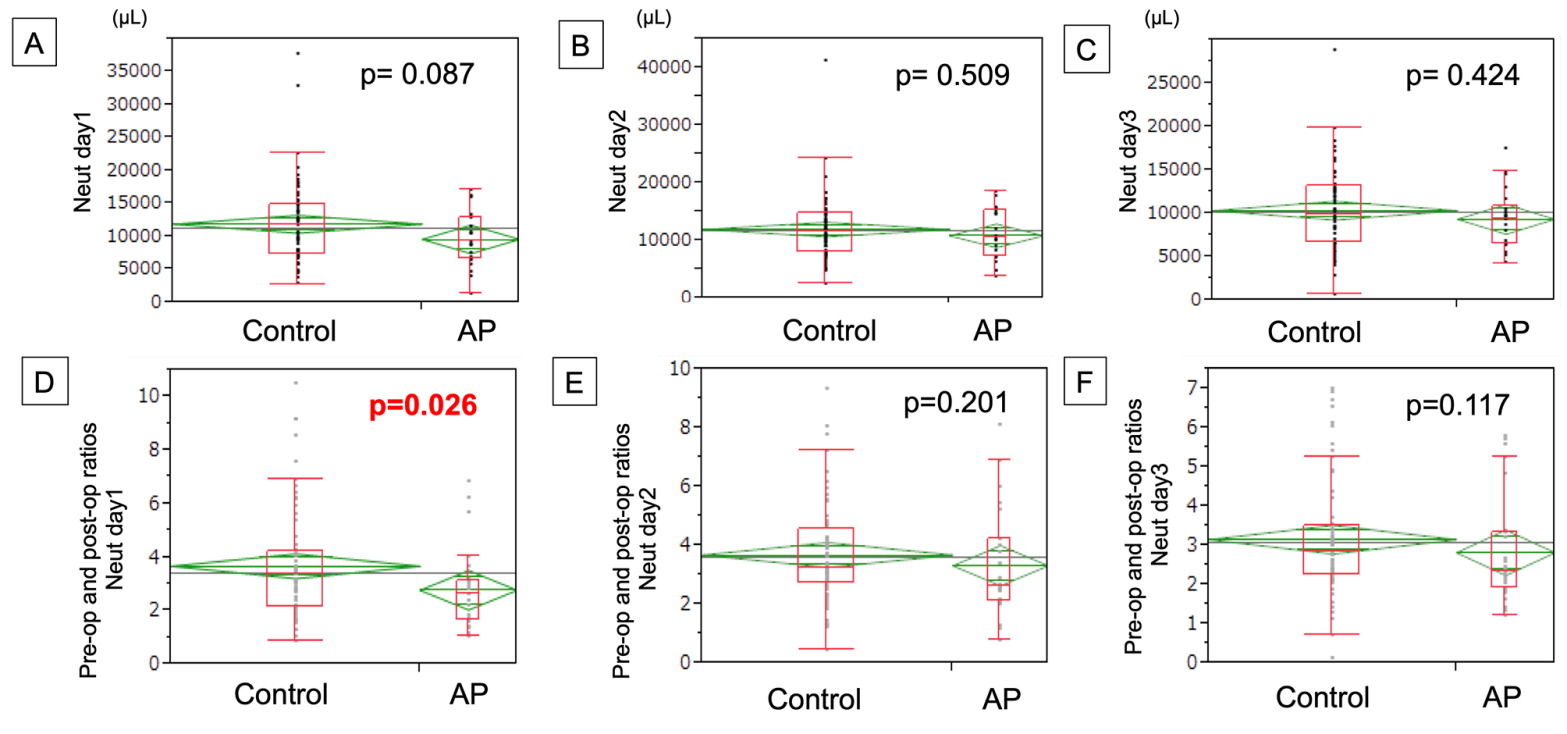
The Neuts count on 1st -3rd POD depending on the use of AP (**A-C**), and the ratio of Neuts where the number of Neuts on each postoperative day was devided by preoperative data (**D-F**)

Figure 4 shows the results regarding Neut. Similar to WBCs, the number of Neut was lower in the AP group (median: 9277.2/µL, range: 1239.7–17080.5/µL) than that in the control group (median: 11823.7/µL, range: 2736–37805.8/µL). The ratio compared by preoperative data (Neut ratio) was also noted to be significantly lower in the AP group than in the control group (*p* = 0.026).

**Fig. 5:**
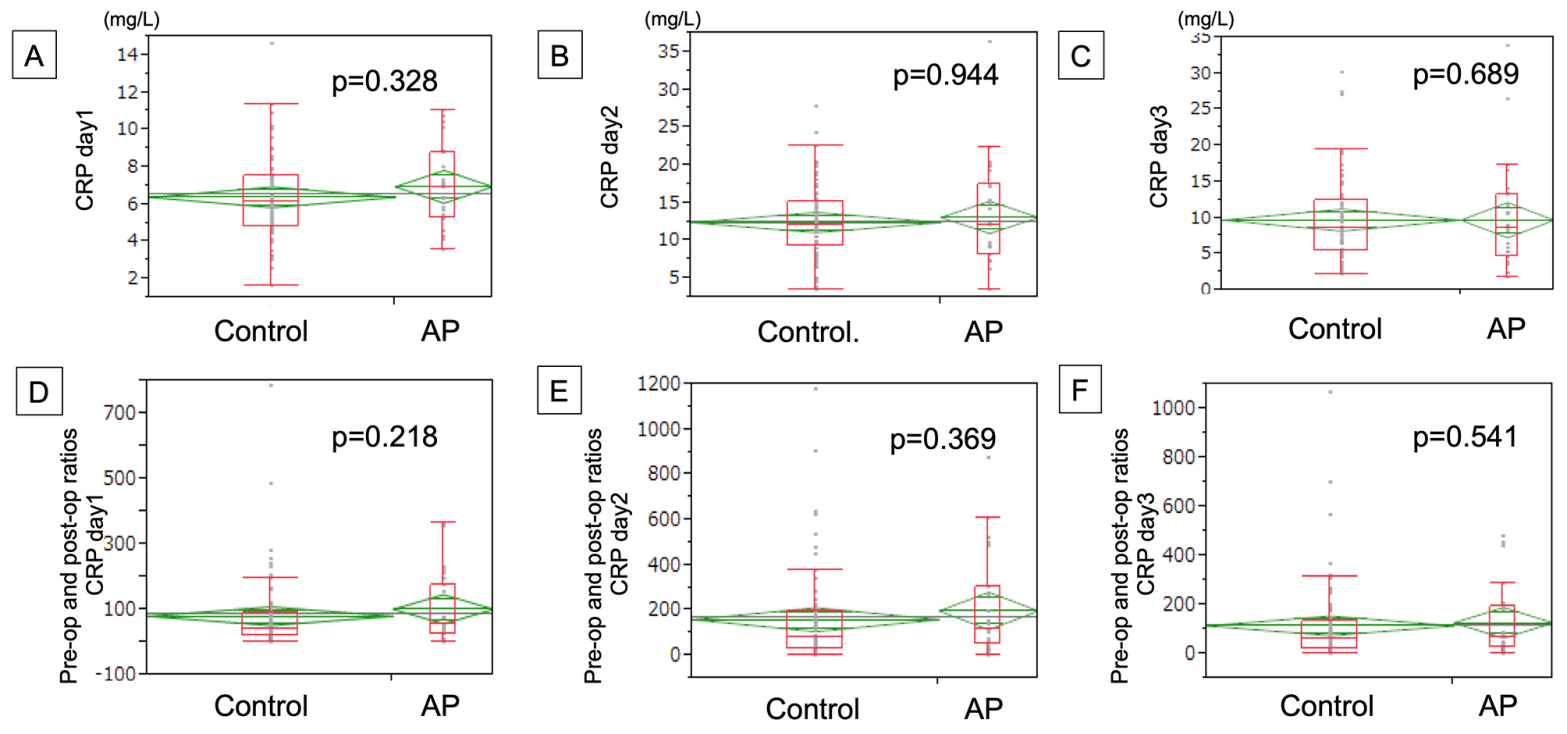
The CRP value on 1st -3rd POD depending on the use of AP (**A-C**), and the ratio of CRP where the number of CRP on each postoperative day was devided by preoperative data (**D-F**)

Figure 5 shows the results of CRP. Contrary to WBC and Neut, no significant differences were found between the AP and control groups, both the measured value and the ratio compared by POD.

### Effect of AP on postoperative complications

The effect of AP on postoperative complications was shown in Table 2. The rate of surgical site infection (SSI) (grade2 or higher) is lower in the AP group (7.4%) than in the control group (20.2%), but with no statistically significant difference (*p* = 0.127). None of the patients in AP group suffered from SSI (grade 3 or higher), while 3 of 69 patients (4.3%) were diagnosed with SSI grade 3 or higher in the control group (*p* = 0.153).

**Table 2:** Percentage of major complications in Grade 0–1 vs. Grade 2–5(C-D classification) in both groups

	AP group (*n* = 27)	Control group (*n* = 69)	*p*-value
Pneumonia			0.6942
present absent	6(22.2%) 21(77.8%)	18(26.1%) 51(73.9%)	
Respiratory complications*			0.3392
present absent	10(37.0%) 17(63.0%)	33(47.8%) 36(52.2%)	
Anastomotic leakage			0.8340
present absent	6(22.2%) 21(77.8%)	14(20.3%) 55(79.7%)	
SSI			0.3977
present absent	1(3.7%) 26(96.3%)	6(8.7%) 63(91.3%)	

* Respiratory complications consist of pleural effusion, pneumothorax

The rate of pneumonia, respiratory complications, and anastomotic leakage was not significantly different between groups in any analyses.

## Discussion

As per our findings, the AP significantly reduced WBC and Neut ratio on POD 1, suggesting its effectiveness in alleviating postoperative excessive inflammation.

Surgical intervention is known to accelerate glycolysis and glycogenesis, decrease insulin sensitivity, and increase inflammatory cytokines [6]. Inflammatory cytokines cause hyperglycemia, which then leads to an increase in cytokines, thereby creating a vicious cycle. Thus, interventions to control postoperative hyperglycemia should be crucial for breaking the cycle [14, 15].

The following important anti-infectious functions of neutrophils are alleviated by hyperglycemia: granulocyte phagocytosis, intracellular bactericidal activity, and immunocompetence. Hence, persistent hyperglycemia has been strongly associated with postoperative infectious complications [16]. Cytokinemia usually increases the number of immune cells because various cytokines are involved in immune cell proliferation. Conversely, cytokine-induced hyperglycemia may not result in an effective increase of immune cells because of their functional problems such as decreased neutrophil migration capacity [17, 18], bactericidal capacity [18–23], and apoptosis [24]. Therefore, the decrease in WBCs and Neut on POD 1 in the AP group can be explained by preventing excess cytokinemia through tight glycemic control (TGC).

Hanasaki et al. reported that TGC targeting IIT between 80 and 110 mg/dl was possible during the perioperative period in patients undergoing hepatectomy, pancreatectomy, and esophagectomy [7, 25–27]. They further reported that stable glycemic control with no hypoglycemic attacks can be obtained in highly invasive hepatobiliary pancreatic surgical patients [28]. Herein, we have determined similar results, which further evidence that AP enables safe glycemic control.

We consider that less inflammation by AP might lead to decrease the infectious complications, although it is not proved in our data set probably because of small number of patients. The effect for complications should be evaluated in larger sample size regarding esophagectomy. Intensive care unit should be appropriate for using AP, so other operative procedures, such as hepatobiliary area, might be good candidates for AP usage.

As for the surgical approach, the percentage of robotic surgery is higher in AP group than in control group. However, the percentage of minimally invasive approach, which includes robotic approach and VATS, is almost same between two groups. Although the advantage of VATS compared with open methos has been clearly shown [29], it still remains controversial whether robot approach is superior to VATS. Thus, we consider that AP group and control group can be fairly compared in terms of the distribution of minimally invasive approach and open method.

As described in the Methods section, we experienced a few patients whose blood glucose levels exceeded 180 despite AP use during the same period. Theoretically, the use of AP should allow for real-time insulin and glucose injection and keep blood glucose levels constant, but we realized that blood glucose values were not always constant. This is mainly because surgeons, nurses, and medical engineers were not familiar with AP usage practically in the early stages of introduction. For example, the venous injection line was not properly inserted during AP usage. We used AP for appropriate blood glucose control after correcting these technical errors.

In this study, AP group with good blood glucose level was compared with the control group which include poor blood glucose level. Thus, we additionally separated the control group into good and poor blood glucose control group. If we defined good control group as patients with blood glucose level less than 180 during ICU stay. 19 patients (27.5%) were categorized as good control group. Notably, this percentage is much less than patients with good control by AP. This data suggests that the quality of blood glucose control can be easily improved by using AP. On the other hand, WBC and Neuts in good control group of standard management is quite similar to AP group (mean WBC on 1POD; 11,348 [AP group] vs. 11,417 [good control by conventional way], mean Neut on 1POD; 9653 [AP group] vs. 9786 [good control by conventional way]). Thus, inflammation can be decreased if blood glucose control is good even by conventional way, but as described, strict control of blood glucose should be more difficult by conventional method than AP.

This is the first study to evaluate AP only in patients who underwent esophagectomy. However, there are some limitations. Although this study showed that AP alleviated early inflammation after esophagectomy, we were not able to observe the effect for reducing postoperative complications. One of the reasons could be the small sample size. Limited term of AP use could be the other reason. In addition, this is a retrospective study conducted by single institute. Thus, we should investigate the optimal use of AP in future study with larger sample size by prospective randomized control study. Furthermore, we need to seek the best candidates for whom AP usage should be beneficial. Another limitation was that inflammatory cytokines were not evaluated; therefore, future studies also should focus on this topic.

In conclusion, we revealed that AP reduces the postoperative inflammatory parameters in patients after esophagectomy. In the future, a large-scale prospective clinical trial is warranted to shed more light on the detailed efficacy of AP.

### Electronic supplementary material

Below is the link to the electronic supplementary material.

Supplementary Material 1

## Data Availability

The datasets used and/or analysed during the current study available from the corresponding author on reasonable request.
