# Supplementary figures and images for: Effects of an artificial pancreas on postoperative inflammation in patients with esophageal cancer
Source: BMC Surg. 2024 Mar 2;24:77. doi: 10.1186/s12893-024-02365-8 (PMC10909248; doi:10.1186/s12893-024-02365-8)

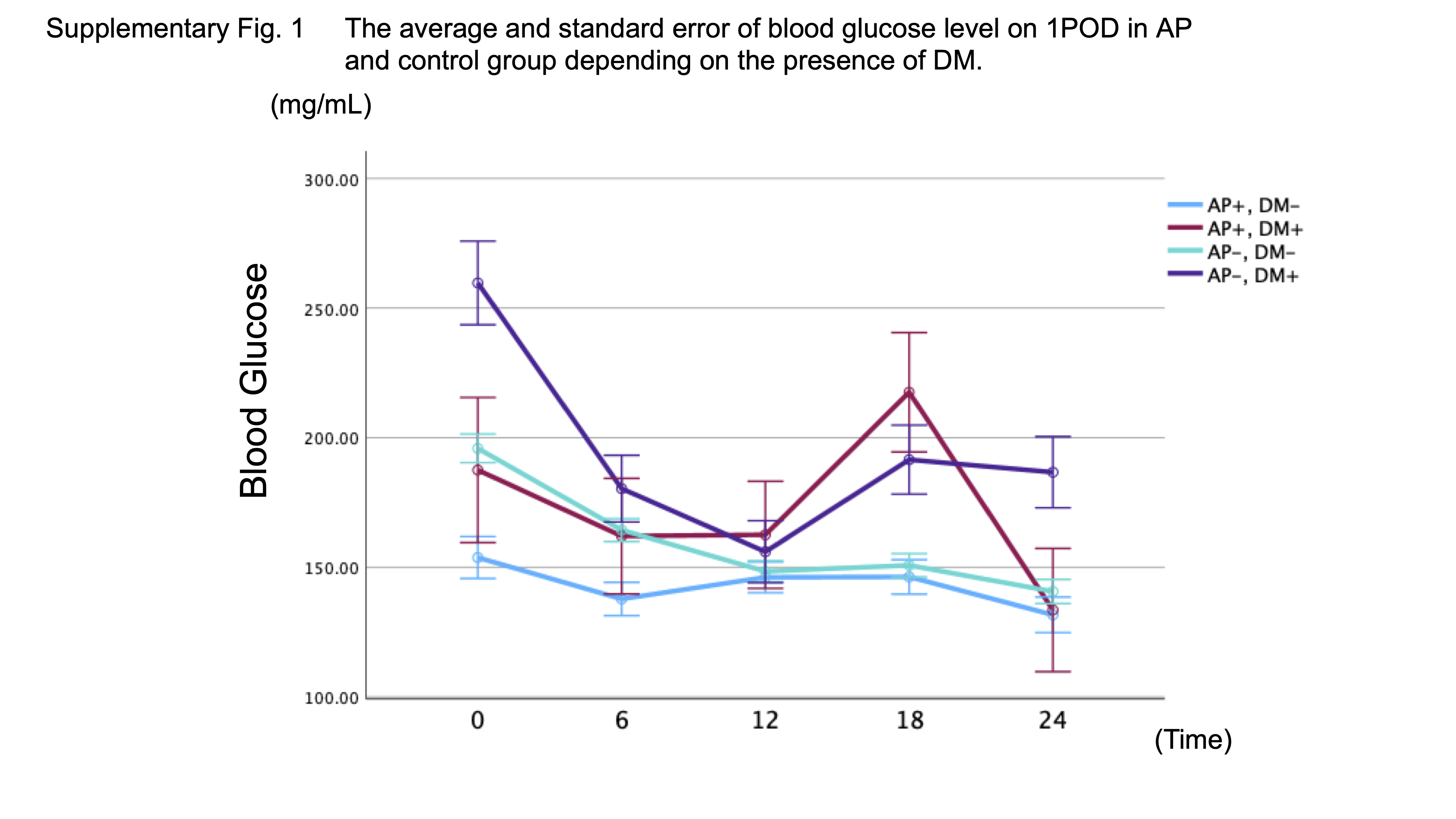

Supplement: Supplementary file 1 — Supplementary Material 1 [file 12893_2024_2365_MOESM1_ESM.tiff]
